# Spectra-based clustering of high-entropy alloy catalysts: improved insight over use of atomic structure†

**DOI:** 10.1039/d4sc06552b

**Published:** 2025-02-10

**Authors:** Huirong Li, Donglai Zhou, Pieter E. S. Smith, Edward Sharman, Hengyu Xiao, Song Wang, Yan Huang, Jun Jiang

**Affiliations:** a State Key Laboratory of Precision and Intelligent Chemistry, University of Science and Technology of China Hefei Anhui 230026 China jiangj1@ustc.edu.cn; b Hefei JiShu Quantum Technology Co. Ltd Hefei 230026 China; c Department of Neurology, University of California Irvine California 92697 USA

## Abstract

The investigation of material properties based on atomic structure is a commonly used approach. However, in the study of complex systems such as high-entropy alloys, atomic structure not only covers an excessively vast chemical space, but also has an imprecise correspondence to chemical properties. Herein, we present a label-free machine learning (ML) model based on physics-based spectroscopic descriptors to study the catalytic properties of AgAuCuPdPt high-entropy alloy catalysts. Even if the atomic structures of two such alloys are different, these alloys may have similar catalytic properties if their spectral characteristics match closely. One cluster with the strongest CO adsorption exhibited high selectivity for C_2+_ product generation, indicating that the spectra-based ML model can provide deeper chemical insight than one based on atomic structure. Moreover, such a model can be extended to other systems with consistent results, thus demonstrating its transferability and versatility. This not only underscores the potential of spectral analysis in identifying high-performance alloy catalysts, but facilitates the formation of a new spectra-based modeling approach and research theory in materials science.

## Abstract 2

Clustering of the spectra of CO adsorbed on high-entropy alloys AgAuCuPdPt, combined with the catalytic properties of each cluster, identified a cluster of high-entropy alloys with high selectivity for C_2+_ products.

## Graphical abstract

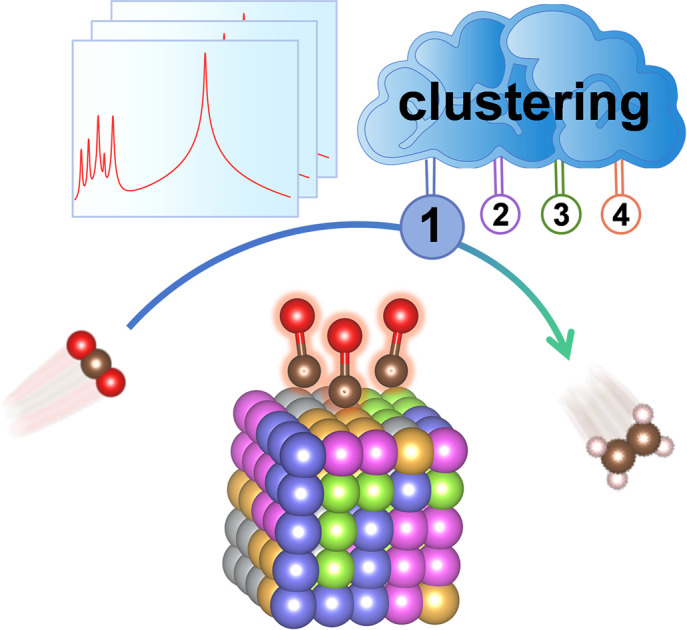

## Introduction

High-entropy alloys (HEAs) are emerging as key players in the field of catalysis, thanks to their distinctive interactions with surface-adsorbed molecules, a factor central to the optimization of catalytic reaction efficacy.^1–3^ The ability to populate their surfaces with a diversity of elements can be leveraged to fine tune their catalytic activity and selectivity.^4–6^ Despite the potential, current research on HEAs tends to focus on improving individual structural aspects.^7–9^ Based on the atomic structure, it is difficult to explore HEAs' vast chemical landscape and identify high-performance catalysts. Because traditional structural descriptors, such as atomic coordinates and elemental types, fall short in comprehensively capturing the diverse influences of adsorption site atoms in adsorbed molecules and neglect crucial electronic information. Spectroscopy is an invaluable tool for integrating experimental and theoretical approaches. It is capable of providing information about not only adsorption energies and adsorption structures but also about the system's electronic structure.^10,11^ This correlation encompasses crucial information about electronic energy levels and charge distribution, endowing spectral signals with the capacity to describe surface–molecule interactions, atomic and chemical bond characteristics, and molecule-chemical environment coupling.

To effectively design HEAs, a comprehensive understanding of their chemical properties is essential, and machine learning has been proven to be an effective tool for extracting insights from spectroscopic data.^12^ It is common practice in computing spectroscopy–property relationships to use supervised learning. However, obtaining reliable labels for supervised learning can be challenging. Unsupervised learning methods, such as cluster analysis, group similar unlabeled sets of data based on their intrinsic properties to uncover patterns and structures that supervised methods may miss.^13^ In particular, spectra-based clustering is a promising method for providing deeper chemical insights into the similarities and differences of HEAs' properties than is attainable by utilizing atomic structure alone.^10,14^

In this study, we employed infrared (IR) vibrational spectroscopy as a key descriptor and applied a clustering model to explore the interactions between adsorbed molecules and high-entropy alloy catalysts, revealing the intricate structure–property relationships in these materials. We focused on HEAs composed of Ag, Au, Cu, Pd and Pt. The noble metals Pd and Pt demonstrate exceptional catalytic activity and stability,^15,16^ while the metals Ag, Au and Cu contribute to enhanced selectivity and adjustability of product distribution.^17^ The similar atomic radii and face-centered cubic lattice structures characteristic of these five metallic elements facilitate the synthesis of AgAuCuPdPt HEAs, resulting in homogeneous solid solutions.^18^

**Fig. 1:**
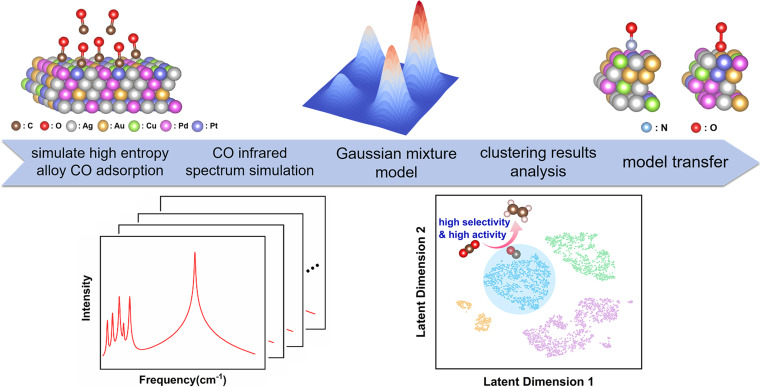
Protocol for using clustering analysis to estimate the catalytic performance of AgAuCuPdPt HEAs using spectroscopic descriptors. Initially, construct various CO adsorption configurations of AgAuCuPdPt HEAs with different compositions and spatial arrangements; subsequently, perform CO IR spectroscopic DFT calculations for each configuration; next, utilize clustering model analysis to analyze the IR spectra and the catalytic characteristics of each cluster; finally, transfer the clustering model to NO and O_2_ adsorption systems.

Therefore, we selected this set of constituent elements, and chose CO as an adsorbate molecule for studying the catalytic performance of HEAs. The characteristics of CO adsorption plays a pivotal role in the carbon dioxide reduction reaction (CO_2_RR), significantly impacting product selectivity.^19–21^ We performed data clustering analysis of IR spectra of CO adsorbed on the surface of AgAuCuPdPt HEAs to obtain distinct clusters, and meticulously analyzed the characteristics of each cluster—including CO adsorption capacity and charge transfer computed by density functional theory (DFT) calculations (Fig. 1). Our results revealed notable differences in the catalytic performance among these clusters. In particular, CO adsorption strength was closely linked to the generation of C_2+_ products. Consequently, the category of vibrational spectrum of a small molecule adsorbed on a new high-entropy alloy can be determined based on the established clustering model (Fig. S4†). The category information can be used to infer the catalytic performance of the high-entropy alloy. This process is implemented through an end-to-end model, which predicts catalytic properties directly from the vibrational spectra, thus eliminating the need for traditional, complex studies of the reaction process and accelerating the screening and development of high-performance catalysts. Furthermore, extending this clustering model to other adsorbed molecules yielded comparable outcomes, suggesting a strong correlation between the spectral data and the catalytic properties of these HEAs.

## Results and discussion

Initially, we constructed a theoretical database of IR spectra and catalytic properties for AgAuCuPdPt HEAs with adsorbed CO, for exploring the intrinsic correlation between CO IR spectra and the CO adsorption process. To obtain additional high-entropy structures, the Monte Carlo method^22^ was applied to randomly generate 4688 configurations of AgAuCuPdPt HEAs with various ratios and atomic structures. In this approach, we first performed DFT structural optimization to obtain catalytic properties including CO adsorption Gibbs free energy and charge transfer for each periodic HEA (Fig. S5†). Since the metal atoms that interacted strongly with CO were mainly located near the adsorption site, we extracted surface metal atoms within 5 Å of the CO molecule for spectral descriptor calculations,^23^ where the coordinates of these metal atoms were fixed (Fig. S6†). Therefore, despite the geometric structure of the local cluster is fixed, metal atoms beyond the 5 Å range influence the coordinates of these fixed atoms. The effect of this broader spatial information can also be reflected to a certain extent in the IR spectra of CO molecules. Furthermore, the number of metal atoms within the 5 Å range typically ranges from 7 to 12, resulting in a combinatorial space of at least 5^7^ = 78 125 possibilities. This makes the combinatorial space of spectral sampling extremely large, encompassing a vast number of possible atomic arrangements, and consequently demonstrating the diversity and intricacy inherent to HEAs. More importantly, these metal atoms exhibit significant interactions with the adsorbed molecules, typically representing the most active sites in catalytic reactions. The relative positions and interactions of these metal atoms can exert a substantial influence on the spectral results. To validate this hypothesis, benchmark tests were conducted on the CO adsorption structures of four typical HEAs. Specifically, a 5 × 5 bilayer HEA metal atom cluster was selected, where the distance between the CO molecule and the farthest metal atom exceeded 5 Å (Fig. S7†). Subsequently, we calculated the CO IR spectra of these structures, with the resulting indicating six distinct vibrational modes, including the CO wagging modes, the HEA–C–O bending modes, the HEA–CO stretching mode, and the C–O stretching mode (Fig. S8†). These spectra were then compared with those obtained from previously smaller single-layer HEA clusters (Fig. S9†). The computational results indicated that the spectra from both methods were highly similar, with Spearman correlation coefficients exceeding 0.91. In particular, the slab thickness has a negligible impact on the frequency, with the frequencies of the characteristic peaks exhibiting near-complete overlap. The influence of atoms beyond a range of 5 Å on the CO spectra is limited. An analysis was also conducted to determine the impact of atomic variations within the 5 Å range on the CO spectra. This analysis involved the comparison of spectra under different conditions, including varying metal types (Fig. S10†), identical metal types but different atomic arrangements (Fig. S11†), and selecting a single spectrum from each cluster to compare its similarity with the remaining spectra in the dataset (Fig. S12†). The resulting Spearman correlation coefficients or their averages were below 0.8, indicating that changes in atoms within the 5 Å range significantly affect the vibrational modes of CO.

Generally, the vibrational modes of CO are typically closely related to their short-range interactions at adsorption sites. However, it is imperative to comprehensively understand the sensitivity of the changes in CO vibrational modes to the surrounding environment of HEAs, the effects of metal atoms with long-range interactions on the vibrational modes of CO must be considered. A comparison was made between the adsorption spectra of CO in HEAs with identical active sites but different surrounding atomic compositions. The results demonstrated significant alterations in the vibrational modes (Fig. S13†). This phenomenon indicates that the vibrational modes of CO can still capture not only the short-range interactions at adsorption sites but also the relatively long-range environmental influences, reflecting the structural diversity of HEAs. Subsequently, the XGBoost algorithm^24^ was applied to assess the significance of vibrational modes in the CO IR spectra (Fig. S14†).^25^ The results indicated that the two lowest-frequency vibrational modes only slightly correlated with the clustering results. Thus, we focused on the other four vibrational modes as key features for characterizing the infrared signals of CO adsorption.

**Fig. 2:**
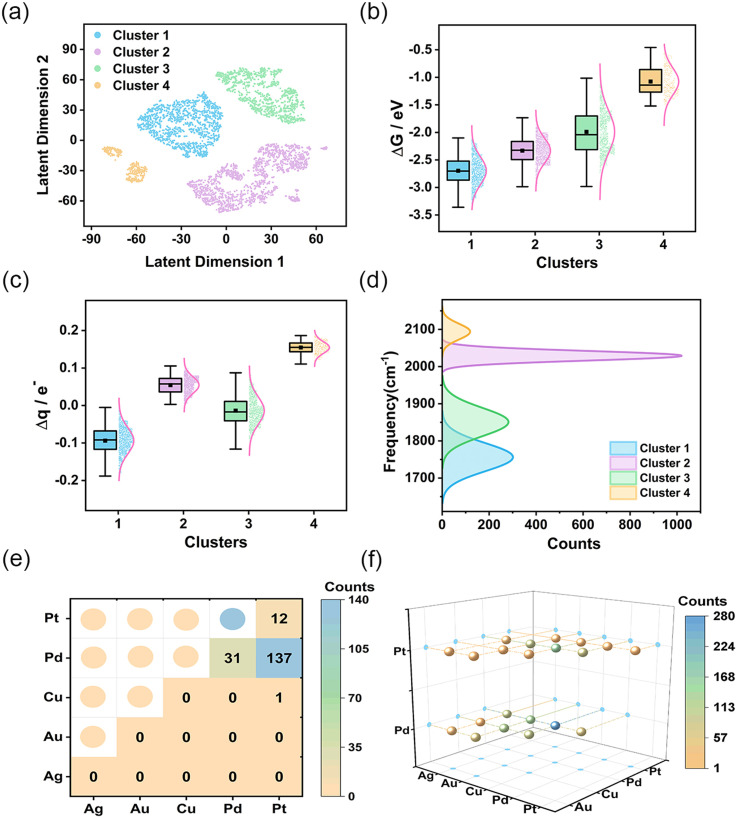
Clustering results of CO IR spectra and analysis of the characteristics of each cluster. (a) Separation of descriptors into four clusters using the t-SNE method. (b and c) Box plots depict the changes in CO adsorption Gibbs free energy and charge transfer for each cluster. (d) Distributions of CO stretching vibration peak frequencies of the four clusters. (e and f) Distribution of bridge and hollow CO adsorption sites on cluster 1 AgAuCuPdPt HEAs.

After defining the spectroscopic feature descriptors, we extracted them from the spectral data. Subsequently, we applied the Gaussian mixture model^26^ to them for feature clustering, resulting in four distinct clusters. To visualize the clustering results, we employed the t-SNE method^27^ to reduce data dimensionality. Unlike conventional linear dimensionality reduction techniques, such as Principal Component Analysis (PCA), the t-SNE method is adept at managing complex nonlinear relationships present in high-dimensional data. The comparison of the results obtained from two-dimensional (2D) and three-dimensional (3D) representations following t-SNE dimensionality reduction (Fig. S15†) indicates that the projections of latent dimension 1 and latent dimension 2 onto the 3D plane closely resemble the 2D visualization. The 2D visualization offers a more intuitive comprehension of the high-dimensional spectra. As shown in Fig. 2a, the four clusters are spatially separated, indicating significant clustering effects. To gain a deeper understanding of the significance of each cluster, we performed a comprehensive assessment of CO adsorption Gibbs free energy and charge transfer for each cluster (Fig. 2b and c). The results demonstrates that AgAuCuPdPt HEAs in cluster 1 exhibited the strongest CO adsorption capacity, coupled with the greatest charge transfer from HEA to CO. HEAs in cluster 2 also exhibited a strong CO adsorption capacity, but with charge being transferred from CO to HEA. In contrast, cluster 3 showed variable CO adsorption performance with a wide range of Gibbs free energies and degrees of charge transfer, revealing the presence of varying adsorption sites. Cluster 4 had the weakest CO adsorption Gibbs free energy, suggesting a limited CO adsorption capacity.

We analyzed the distribution of the four vibrational frequencies and intensities utilized in each cluster (Fig. S16†). As illustrated in the figure, the overlap of intensities within the clusters is considerable, suggesting that intensity is not the primary factor differentiating the clusters. In contrast, the boundaries of the frequencies are much more distinct, particularly for the first and fourth clusters, where the intervals between each frequency are the widest. This indicates that the adsorption structures and catalytic performance of these substances differ the most and the distribution of frequencies plays a pivotal role in the clustering of the data. Our analysis was therefore focused on the CO adsorption frequency, especially the prominent stretching vibration peaks located in the high-frequency IR region.^28^ Notably, this peak occurred at the lowest frequency in cluster 1 HEAs, while in cluster 4 HEAs, it occurred at the highest (Fig. 2d). This result underscores the direct correlation between CO adsorption strength and shift in the vibration peak. Subsequently, the projected density of states (PDOS) of the adsorption structure was calculated using DFT to comprehensively analyze the adsorption behavior and electronic structure characteristics of CO on the HEA surface (Fig. S17†). As illustrated in the figure, the charge density of HEAs is much higher than that of CO. There is an overlap between the orbitals of the high-entropy alloy and the molecular orbitals of CO, indicating an interaction between the CO molecule and the HEA. Specifically, the metallic properties of HEAs exert a considerable influence on the electronic structure of the adsorbed CO, resulting in a modification of the strength of the C–O bond and, consequently, the vibrational frequency. In particular, if the adsorbed metal site exerts a strong electron-donating effect, the C–O bond will undergo elongation and weakening due to the intense electron filling, resulting in a notable decrease in the C–O vibrational frequency. Conversely, if the electron-donating effect of the adsorbed metal site is weak, the C–O bond will retain a vibrational frequency that is similar to that of a free CO molecule. Furthermore, we conducted an analysis of the factors contributing to the alterations in other vibration modes. HEA–C–O bending vibration (modes 3 and 4) reflect changes in geometric and surface electronic structures during the adsorption of CO (Fig. S16a and b†). In cluster 1, the interaction between the CO π orbitals and the metal orbitals causes a redistribution of the electron density within the CO molecule. This redistribution strengthens the interaction between the C–O bond and the surface metal atoms, thereby increasing the vibrational frequency. In the stretching vibration of HEA–CO (mode 5), the focus is on the covalent interactions between the CO molecule and the HEA surface (Fig. S16c†). In cluster 1, strong adsorption and electron donation from the surface metal atoms to CO enhance the chemical bonding between the alloy and CO, resulting in an increased vibrational frequency. In contrast, the cluster 4 demonstrates weaker interactions, leading to a reduced vibrational frequency. This demonstrates that spectral analysis is able to precisely define structural properties, enabled by its high degree of sensitivity to variations in the data achieved by the clustering method. This sensitivity is crucial for discerning subtle differences in the catalytic performance of the HEAs, thereby providing a comprehensive depiction of an alloy's interaction with CO at a molecular level. The connection between the spectra and the catalytic properties also enhances the interpretability of the clustering results.

We further analyzed the adsorption environments in the clusters, particularly noting the structural features unique to cluster 1. This cluster was characterized by two primary adsorption modes: hollow adsorption, identified in 1289 instances, and bridge adsorptions, observed in 181 cases (Fig. S18†). Fig. 2e and f illustrates the distribution of these two adsorption sites. Bridge adsorption sites were predominantly concentrated on Pd and Pt atoms, while the atoms creating hollow adsorption sites were more diverse, covering atoms of all five metals observed, with Pd, Cu, Au, and Pt being more abundant (Fig. S19†). The presence of these active sites enhances the adsorption of CO on the alloy surface, while also promoting the flow of electrons to CO, strengthening the connection between HEAs and C, weakening the C–O bond and causing a decrease in the stretching vibration frequency of CO. In clusters 2 and 4, the top adsorption mode was exclusively observed; this mode reduced adsorption energies and encouraged CO molecules to release electrons, which leads to an increase in the C–O vibration frequency. The experimental spectra of CO align with the theoretical spectra, showing that the stretching vibration frequency of the C–O bond decreases in bridge adsorption compared to linear adsorption, which results in a redshift.^29,30^ In cluster 2, the adsorption sites were primarily at Pd and Pt (Fig. S20†), while Ag and Au acted as the key adsorption sites in cluster 4 (Fig. S21†). In Fig. 2a, cluster 2 data appear in the lower right (violet points), while cluster 4 data are positioned in the lower left (orange points). The clean separation of these distributions distinctly validates the proficiency of the clustering technique in identifying and differentiating between diverse adsorption environments. Cluster 3 emerged as the most varied, with a mix of three adsorption modes including 3 top adsorptions, 555 bridge adsorptions, and 589 hollow adsorptions (Fig. S22–S24†). This cluster incorporated all five metals, with Cu being the predominant element, followed by Au, Pd, Ag, and Pt in descending order of abundance (Fig. S25†). The unique feature of this cluster displayed the broadest range of data distribution and energy adsorption variation. In addition, a comparative analysis of the PDOS under varying adsorption sites and modes was conducted, as illustrated in Fig. S26.† The investigation revealed that the charges of Pd and Pt exhibit enhanced concentration and intensity in the vicinity of the Fermi level and the coupling peaks of the PDOS of CO near the Fermi level are more pronounced, suggesting a robust adsorption interaction of CO at these metal sites. Conversely, the PDOS of Ag and Au near the Fermi level exhibits a paucity of peaks, with a significant reduction or near absence of coupling peaks in the CO PDOS, indicative of weaker CO adsorption at these sites. Then we constructed a distribution plot for the structures and adsorption energies of each adsorption site within each cluster (Fig. S27†), which also indicated the strength of adsorption is closely related to the metal composition of the adsorption sites. As the content of Pd and Pt at the adsorption sites increases, the adsorption becomes stronger; conversely, an increase in Ag and Au content weakens the adsorption. The variety of adsorption modes and their distribution across different metal compositions not only reflect the unique catalytic potentials of each cluster, but also highlight the intricate interplay of structural and electronic factors in determining catalytic efficacy.

**Fig. 3:**
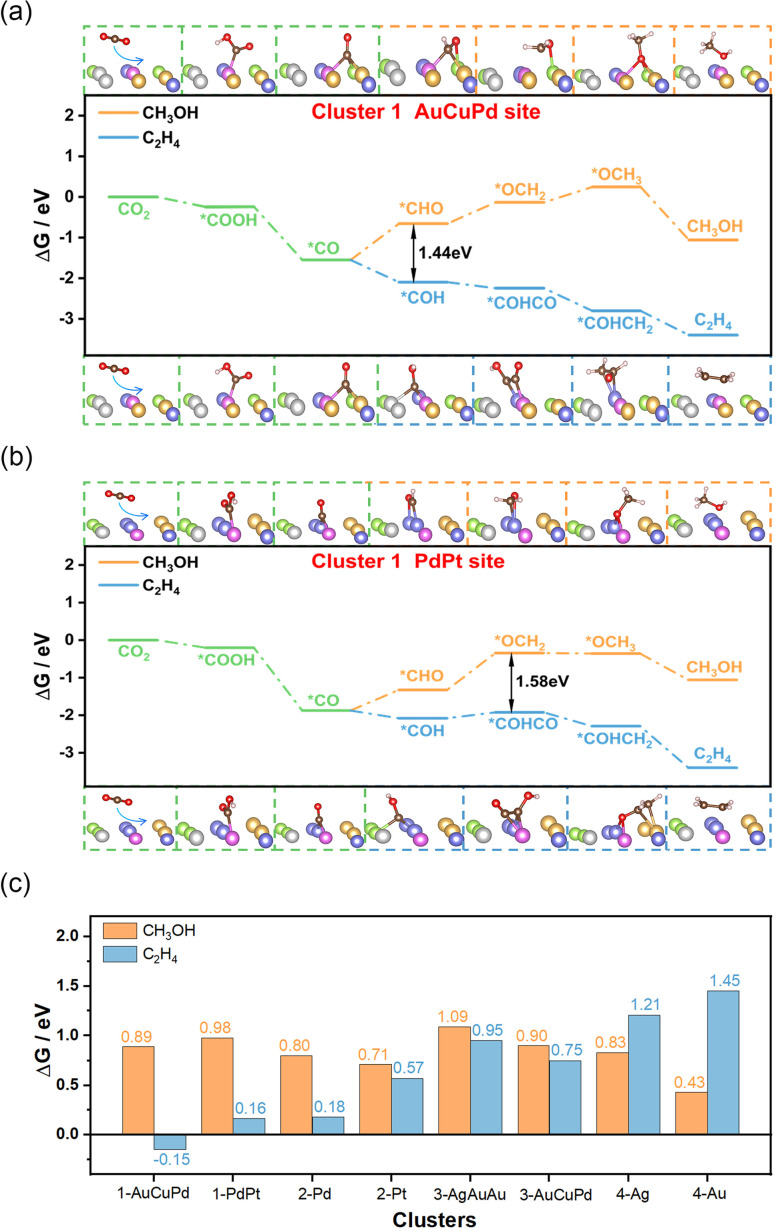
Validation calculations of AgAuCuPdPt high-entropy cluster 1 alloy structures that demonstrate high selectivity and activity for C_2+_ products of the CO_2_RR. (a) Free energy diagram for the production of CH_3_OH and C_2_H_4_ on the hollow site composed of AuCuPd metal atoms in cluster 1. (b) Free energy diagram for the production of CH_3_OH and C_2_H_4_ on the bridge site composed of PdPt metal atoms in cluster 1. (c) Comparison of the free energy barriers of the RDS for the production of CH_3_OH or C_2_H_4_ in all clusters.

When adsorption of CO is enhanced, the CO residence time on the catalyst is prolonged, thus increasing the possibility of C–C coupling and facilitating the generation of C_2+_ products.^31–33^ Additionally, experimental evidence suggests a correlation between the C–O stretching vibration frequency and catalytic performance. The strong interaction between CO and the catalyst weakens the C–O stretching vibrations and reduces the energy barrier associated with coupling.^34,35^ The HEAs in cluster 1 exhibited the strongest adsorption capacity for CO and the weakest C–O stretching vibrations, making them the most likely candidates for the production of C_2+_ products. To confirm this hypothesis, we randomly selected two structures from cluster 1 with distinct adsorption sites composed of AuCuPd or PdPt, and computed the entire process of creating C_2_H_4_ and CH_3_OH (Fig. 3a and b). The outcome revealed that generating CH_3_OH had a considerably higher energy barrier than producing C_2_H_4_. This suggested that cluster 1 catalysts have a pronounced preference for generating C_2+_ products. The lower energy barrier for C_2_H_4_ formation underlines the nuanced catalytic behavior of these alloys, indicating a potential for tailored catalysis of the CO_2_RR. For the remaining clusters, we also randomly selected several structures and analyzed their catalytic behavior. In cluster 2, two structures with Pd and Pt adsorption sites for CO were examined, and both predominantly favored the production of C_2_H_4_ compared to the generation of CH_3_OH (Fig. S28 and S29†). In cluster 3, although the hollow sites composed of AgAuAu or AuCuPd were slightly more likely to generate C_2_H_4_ products, the tendency was not obvious (Fig. S30 and S31†). As for cluster 4, the Ag or Au sites within the high-entropy alloy catalysts were entirely favorable for producing CH_3_OH (Fig. S32 and S33†). Most notably, cluster 1 exhibited the lowest free energy barrier of the rate-determining-step (RDS) for producing C_2_H_4_ compared to other clusters, indicating its enhanced catalytic activity for generating C_2+_ products (Fig. 3c). These findings highlight the unique catalytic performance of each cluster and demonstrate that cluster 1 exhibits high selectivity and activity for C_2+_ products. This insight into the specific catalytic behavior of cluster 1 opens up new avenues for the development of specialized high-entropy catalysts.

**Fig. 4:**
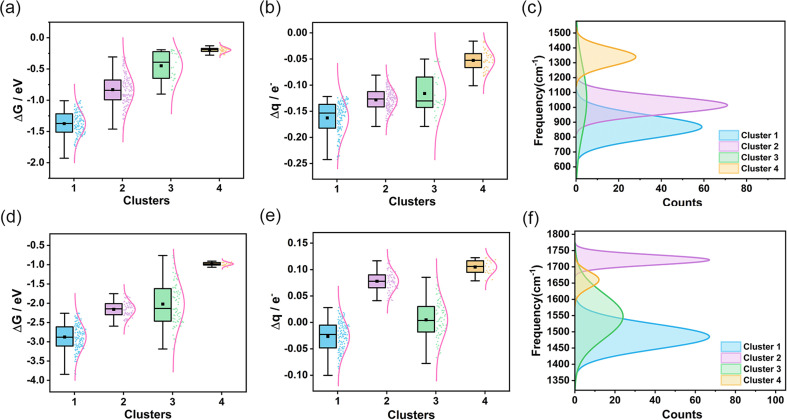
Clustering of O_2_ and NO IR spectra and analysis of the characteristics of each cluster. (a and b) Box plots depict the changes in O_2_ adsorption Gibbs free energy and charge transfer for each cluster. (c) Distribution of O_2_ stretching vibration peak frequencies for the four clusters. (d and e) Box plots depict the change in NO adsorption Gibbs free energy and charge transfer for each cluster. (f) Distribution of NO stretching vibration peak frequencies for the four clusters.

To assess the transferability and versatility of the spectral-based clustering model, we extended our analysis to other diatomic molecules (Fig. S34 and S35†) and aimed to determine if these small molecules exhibit comparable characteristics in our clustering framework. We constructed more limited data for O_2_ and NO, calculating the spectral and catalytic performance of 359 AgAuCuPdPt HEAs adsorbing O_2_ and 300 AgAuCuPdPt HEAs adsorbing NO. Remarkably, when applying the same clustering model used for CO, we observed analogous clustering outcomes (Fig. S36 and S37†). From the IR spectra of CO, O_2_, and NO molecules adsorbed on the high-entropy alloy surface (Fig. S38†), it can be observed that these small molecules exhibit similar bending vibrational modes in the low-frequency region and similar stretching vibration peaks in the high-frequency region. These commonalities facilitate comprehension of the adsorption behavior and vibrational characteristics of diverse small molecules on high-entropy alloy surfaces. We noted that in both adsorbing O_2_ and NO scenarios, the interaction strength between the high-entropy alloy surface and the small molecule adsorbate is most pronounced in cluster 1, diminishing progressively in the other three clusters (Fig. 4a and d). Furthermore, our analysis revealed a persistent correlation between the degree of charge transfer from the small molecule to the high-entropy alloy and the frequency of the stretching vibrational peak. Specifically, greater charge transfer from HEA to O_2_ or NO correlated with lower frequency vibrational peaks, suggesting that the spectral data reliably reflects the adsorption state of the small molecules on the HEAs (Fig. 4b, c, e and f). A further analysis of the metal composition at the adsorption sites for CO, O_2_, and NO in each cluster (Fig. S39†) revealed that the overall metal composition is quite similar. The first cluster exhibits elevated concentrations of Cu, Pd, and Pt, whereas the fourth cluster displays augmented levels of Ag and Au. The alterations in metal composition at the adsorption sites were corroborated by IR spectroscopy, wherein the vibrational characteristic peaks exhibited regular shifts. These trends align with our earlier findings for CO adsorption, illustrating the robustness of our clustering model. This similarity in results across different molecules not only validates the transference of the clustering model but also emphasizes its versatility in categorizing various molecular adsorptions. The consistent patterns we identified suggest that our model can effectively discern and classify adsorption characteristics, regardless of the specific molecule involved. This finding highlights the potential of our methodology to serve as a powerful tool in the broader realm of molecular spectroscopic analysis.

## Conclusions

This study conducts a significant exploration into the catalytic capabilities of AgAuCuPdPt HEAs through a spectra-based clustering model. The interactions between these HEAs and various adsorbed molecules were captured effectively by computing and analyzing the IR spectra generated by vibrational modes of the adsorbed species. Properties of these systems were found to cluster, permitting us to elucidate distinct catalytic properties of each cluster. Notably, the strong CO adsorption characteristic of cluster 1 significantly enhances the likelihood of producing C_2+_ products. It is also interesting to find that HEAs can have similar catalytic selectivities even though they possess different atomic structures, suggesting that spectroscopic analysis is a better tool to describe material properties. This study provides invaluable insights for the design and development of efficient and specialized high-entropy alloy catalysts. It also explores an entirely new path towards establishing a spectroscopic encoding method for chemical substances, as it is a spectra-based chemical theory that does not depend on structural coordinates. This approach could be expanded in the future to obtain more promising catalysts by exploring other types of alloys and alternative spectroscopic techniques, particularly those derived from dynamic simulations that are more representative of real-world environments.

## Data availability

All codes and datasets used in this paper are summarized at https://github.com/lihr1008/high-entropy-alloys.git.

## Author contributions

H. L. and D. Z. contributed equally to this work. H. L. and D. Z. carried out all the theoretical calculations, which involved DFT and AIMD simulations, to construct the dataset for the machine learning model. Y. H., H. L., D. Z. and H. X. utilized the machine learning model to perform a comprehensive analysis of the dataset. J. J., Y. H., S. W., H. L., D. Z., P. E. S. S. and E. S. co-wrote the paper. J. J., Y. H. and S. W. conceived the idea and supervised the project. All authors discussed the results and commented on the manuscript.

## Conflicts of interest

There are no conflicts to declare.

## Supplementary Material

SC-OLF-D4SC06552B-s001
