# Supplementary material for: Spectra-based clustering of high-entropy alloy catalysts: improved insight over use of atomic structure
Source: Chem Sci. 2025 Feb 10;16(11):4646–53. doi: 10.1039/d4sc06552b (PMC11808395; doi:10.1039/d4sc06552b)
Supplement: SC-016-D4SC06552B-s001 [file SC-016-D4SC06552B-s001.pdf]

Supplementary Information (SI) for Chemical Science.
This journal is © The Royal Society of Chemistry 2025

Supplementary Information for:

Spectra-Based Clustering of High-Entropy Alloy Catalysts: Improved Insight Over Use of Atomic Structure

Huirong Li[a#], Donglai Zhou[a#], Pieter E. S. Smith[b], Edward Sharman[c], Hengyu Xiao[b], Song Wang[a*], Yan Huang[a*], Jun Jiang[a*]

[a] State Key Laboratory of Precision and Intelligent Chemistry, University of Science and Technology of China, Hefei, Anhui 230026, China

[b] Hefei JiShu Quantum Technology Co. Ltd., Hefei 230026, China

[c] Department of Neurology, University of California, Irvine, California 92697, USA

Methods

Acquisition of high-entropy alloy structures. The atomic structures of high-entropy alloys (HEAs) were generated using the Alloy Theoretic Automated Toolkit (ATAT) software[1, 2]. The software employs a Monte Carlo algorithm to identify Special Quasirandom Structures (SQS), thereby accurately representing the complex, multicomponent nature of HEAs.

The process for generating HEAs is as follows:

(1) Initial Structure Setup. The (111) plane of a face-centered cubic (fcc) lattice alloy is utilized as the preliminary input file. A unit cell containing four layers of atoms is created, with each layer arranged in a 3×3 matrix. The aforementioned file incorporates a comprehensive array of parameters, including the lattice vectors, the lattice angles, supercell parameters in the x, y, and z directions, and atomic coordinates.

(2) Element Composition Generation. The composition ratio of each element in the structure is randomly generated based on the ratio of the number of atoms to the number of surface atoms. The proportion information of each metal is incorporated into the input file, and the lattice constants are modified through a weighting process that is contingent upon the metal proportions.

(3) The definition and constraints of clusters. Consider clusters formed by any two atoms within a distance of 6 units, and restrict the pairing length between ternary and quaternary clusters to be less than 5.2 units.

(4) Monte Carlo Simulations. The supercell multipliers in all directions are set to 1 and written to sqscell.out. The command mcsqs -rc -n=36 is then executed, employing the Monte Carlo method to progressively alter the elemental configuration of the alloy.

(5) Structure Generation. During the simulation, mcsqs perpetually explores diverse atomic arrangements until the system converges to a stable configuration or the predefined computational conditions are met, thereby generating a stable alloy structure. This configuration is characterized by a reduction in energy and a more uniform atomic arrangement (Fig. S1).

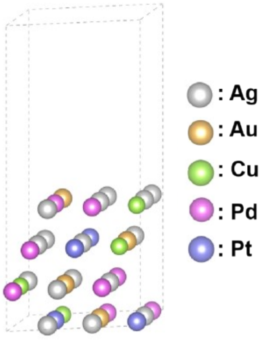

Fig. S1. The configuration of AgAuCuPdPt high-entropy alloy.

DFT calculations. The initial adsorption configuration for each HEA was selected by placing the small molecule adsorbate directly above the central position of the HEA surface, followed by DFT structure optimization[3]. The structure of the small molecule and the surface metal atoms in the high-entropy alloy were relaxed. All DFT calculations for the HEAs were performed using the Vienna Ab-initio Simulation Package (VASP)[4]. To account for long-range van der Waals (vdW) interactions, Grimme's DFT-D3[5] method was employed, with an energy cut-off set at 400 eV. The

Perdew-Burke-Ernzerhof (PBE) functional[6] was utilized, and the Projector Augmented Wave (PAW) method[7] served as the basis set for plane-wave expansion. For geometric optimization of the HEAs, convergence criteria for forces and energies were set to 0.01 eV/Å and 10^-5 eV, respectively. Brillouin zone sampling was carried out using a 1 × 1 × 1 Monkhorst-Pack k-mesh, alongside a vacuum space of approximately 10 Å.

IR spectra calculations. Infrared (IR) calculations were performed using Gaussian 16[8]. For these computations, the PBE0 hybrid functional[9] was employed, coupled with the 6-31+G* basis set[10] for main-group elements. Additionally, the pseudo LANL2DZ basis set[11] was used for the metal atoms. Static calculations facilitate a more direct analysis of interatomic interactions and provide a valuable baseline for comparative analysis with more complex dynamic behaviors[12]. Additionally, static calculations require fewer computational resources, enabling systematic exploration across the extensive configuration and compositional space of HEAs. To provide further validation of the impact of dynamic atomic behavior on catalytic performance, additional ab initio molecular dynamics (AIMD) simulations were conducted with the aim of studying the adsorption process of CO molecules on different HEA structures. In the AIMD simulations, the system was first heated from 0 K to 300 K within 2 ps and then maintained at 300 K for 5 ps to achieve thermodynamic equilibrium, thereby obtaining stable adsorption structures (Fig. S2). The analysis results indicate that the static calculation method accurately identifies the majority of CO adsorption sites. Despite the fact that the adsorption energies derived from AIMD calculations are slightly lower than those obtained from static calculations due to temperature effects, this discrepancy does not have a significant impact on our overall evaluation of the adsorption behavior. Furthermore, to simulate the dynamic process of CO adsorption on HEAs at 300 K in a more comprehensive manner, the AIMD simulation time was extended to 10 ps. The coordinates, velocities, and dipole moment variations of the CO molecules during the simulation were extracted, and the MLatom method was employed to construct the dynamic spectra of CO[13]. A comparative analysis with the static spectra was conducted (Fig. S3). The outcomes of this study indicate that the dynamic spectra demonstrate heightened sensitivity in the low-frequency vibrational modes, with peak shapes that more closely resemble the irregular fluctuations observed in experiments. This finding suggests that dynamic simulations may offer a more precise representation of the vibrational characteristics present in actual environments. In the high-frequency region, the characteristic peak frequencies of the dynamic spectra closely align with those obtained from static spectra calculations. High-frequency vibrations of CO directly reflect the strength of adsorption and charge transfer between CO and the HEAs, which are highly correlated with the catalytic performance of the HEAs. Consequently, the conclusions derived from static spectra are considered reliable.

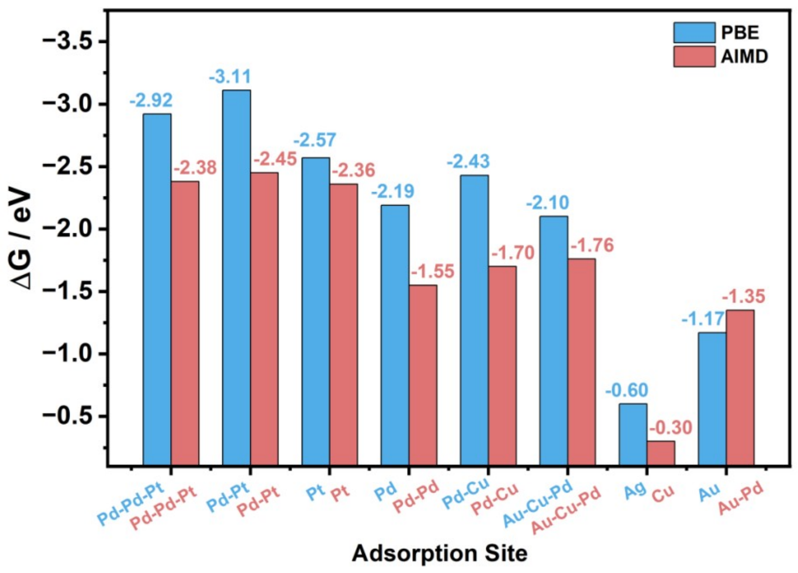

Fig. S2. Comparison of adsorption sites and energies calculated using the PBE functional and AIMD.

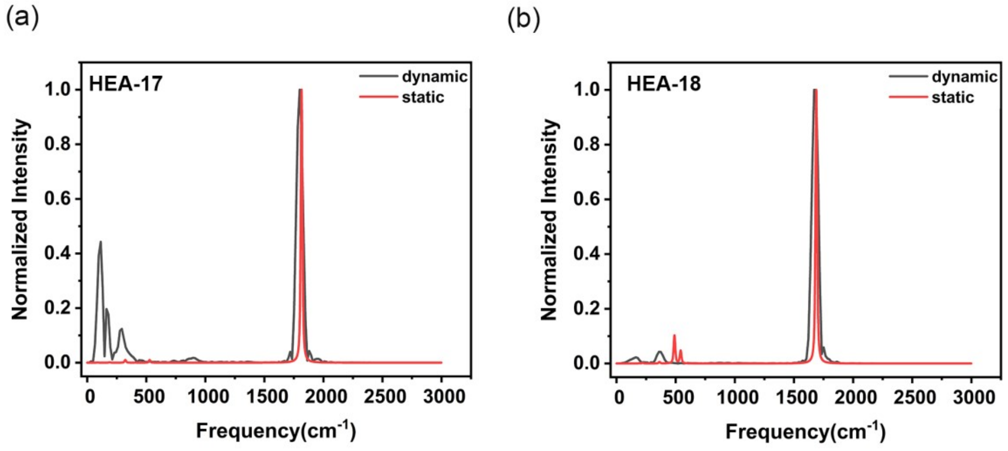

Fig. S3. Comparison of static and dynamic spectra.

Free Energy and Charge Calculations.

The elementary steps for generating CH_3OH can be given as[14]:

$$*+CO_2(g) + H^+ + e^- \rightarrow *COOH \quad (1)$$

$$*COOH + H^+ + e^- \rightarrow *CO + H_2O(l) \quad (2)$$

$$*CO + H^+ + e^- \rightarrow *CHO \quad (3)$$

$$*CHO + H^+ + e^- \rightarrow *OCH_2 \quad (4)$$

$$*OCH_2 + H^+ + e^- \rightarrow *OCH_3 \quad (5)$$

$$*OCH_3 + H^+ + e^- \rightarrow CH_3OH(l) \quad (6)$$

The elementary steps for generating C_2H_4 can be given as[15]:

$$* +CO_2(g) + H^+ + e^- \rightarrow *COOH \quad (1)$$

$$*COOH + H^+ + e^- \rightarrow *CO + H_2O(l) \quad (2)$$

$$*CO + H^+ + e^- \rightarrow *COH \quad (3)$$

$$*COH + CO + * \rightarrow HOC**CO \quad (4)$$

$$HOC**CO + 4H^+ + 4e^- \rightarrow HOC**CH_2 + H_2O(l) \quad (5)$$

$$HOC**CH_2 + 3H^+ + 3e^- \rightarrow C_2H_4(g) + H_2O(l) \quad (6)$$

Standard Gibbs free energy changes at zero potential were calculated using the following formula:

$$\Delta G = \Delta E + \Delta ZPE - T\Delta S$$

In this equation, ΔE represents the change in reaction energy as determined by DFT simulations. ΔZPE is the change in zero-point energy, calculated using the vibrational frequencies obtained through the finite difference method. ΔS denotes the entropy change for each elementary step. For our calculations, the temperature was set at 298.15 K. Additionally, atomic charges were computed employing the Density-Derived Electrostatic and Chemical (DDEC06) atomic population analysis method[16].

Clustering model. In this study, we employed the Gaussian mixture method (GMM) for cluster analysis of spectral data. GMM is a probabilistic clustering method that operates under the assumption that the data are generated from a mixture of multiple Gaussian distributions with unknown parameters[17]. Each cluster corresponds to a specific Gaussian distribution, and GMM identifies potential clustering structures by modelling the probability density of the data points. The method is characterized by an iterative process in which the means, variances, and weights of each distribution are estimated using the Expectation-Maximization (EM) algorithm. In each iteration, the algorithm first calculates the probability of each data point belonging to each cluster (the Expectation step), and then updates the parameters of the Gaussian distributions to maximize these probabilities (the Maximization step). We categorized the data into four distinct groups and set the covariance type to 'spherical'. The process involved four initializations, and iterations were conducted using the EM algorithm[18]. Iteration was halted when the stopping threshold reached 1e-3, and the maximum number of iterations was set to 100.

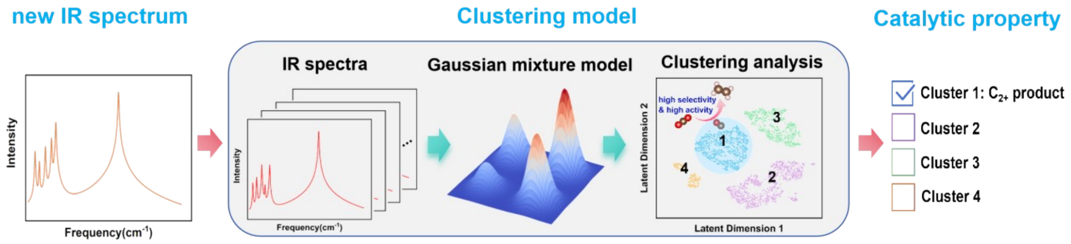

Fig. S4. Flowchart for identifying vibrational spectra of a small molecule adsorbed on a new high entropy alloy using clustering models.

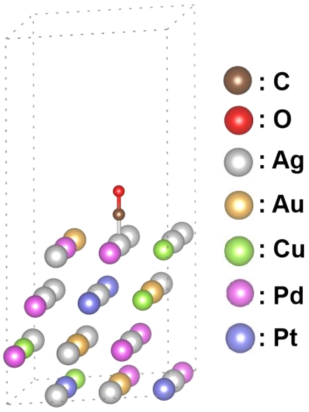

Fig. S5. The initial configuration of CO adsorption on the AgAuCuPdPt high-entropy alloy surface.

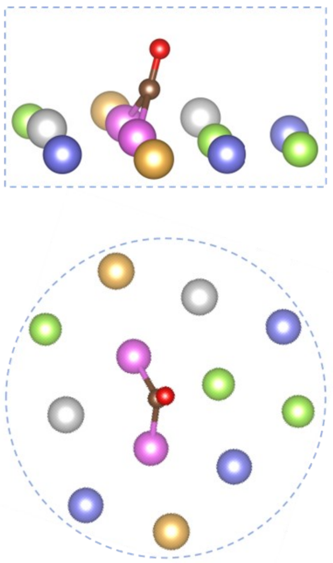

Fig. S6. Configuration of metal atom aggregates within a 5Å radius of the adsorbed CO molecule.

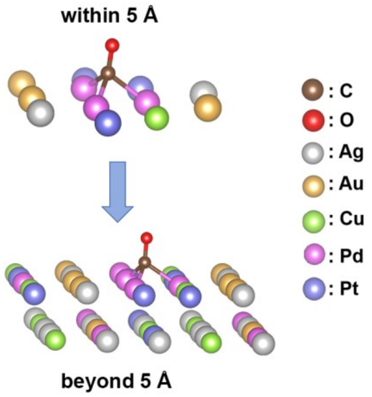

Fig. S7. A schematic diagram illustrating the expansion from a single-layer atomic cluster within 5 Å to a double-layer atomic cluster beyond 5 Å.

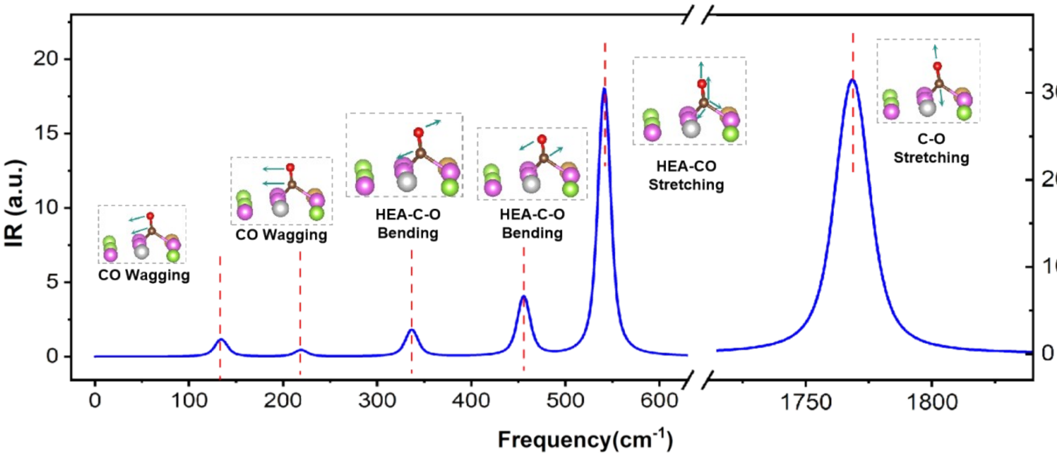

Fig. S8. Spectra and schematic diagrams of the six vibrational modes of CO adsorbed on the surface of the AgAuCuPdPt high-entropy alloy.

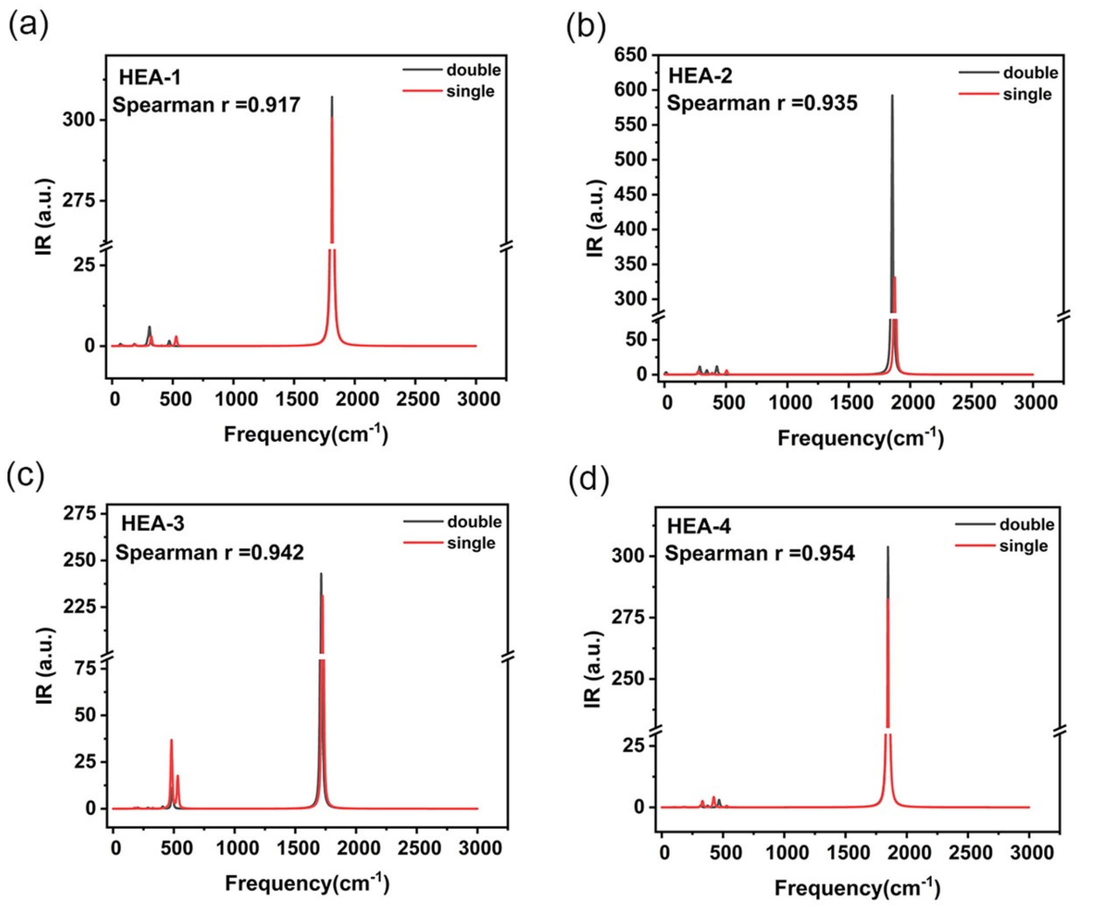

Fig. S9. (a)-(d) represent the comparison of infrared spectra calculated for single-layer metal surfaces within 5 Å and double-layer metal surfaces exceeding 5 Å with CO adsorption on four different HEAs, HEA-(1-4).

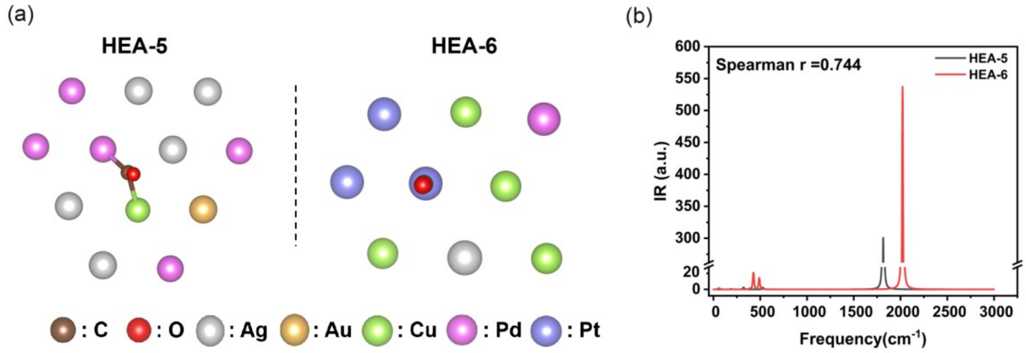

Fig. S10. Comparison of CO spectra for different types of metals within 5 Å in HEA-5 and HEA-6.

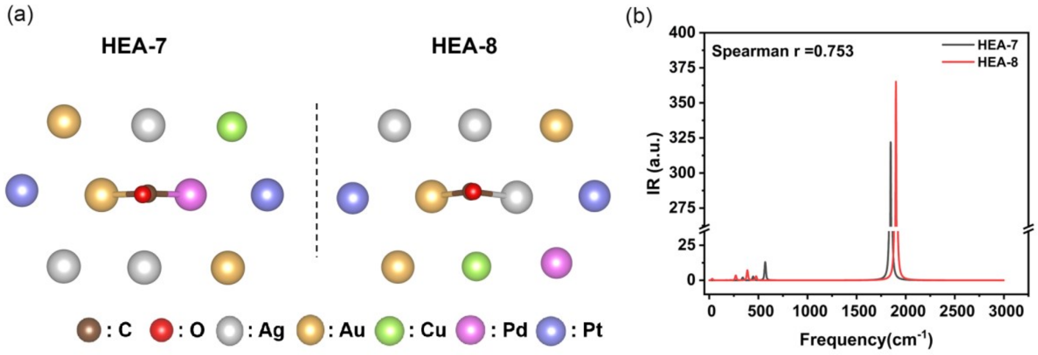

Fig. S11. Comparison of CO spectra for identical types of metals within 5 Å but different metal arrangements in HEA-7 and HEA-8.

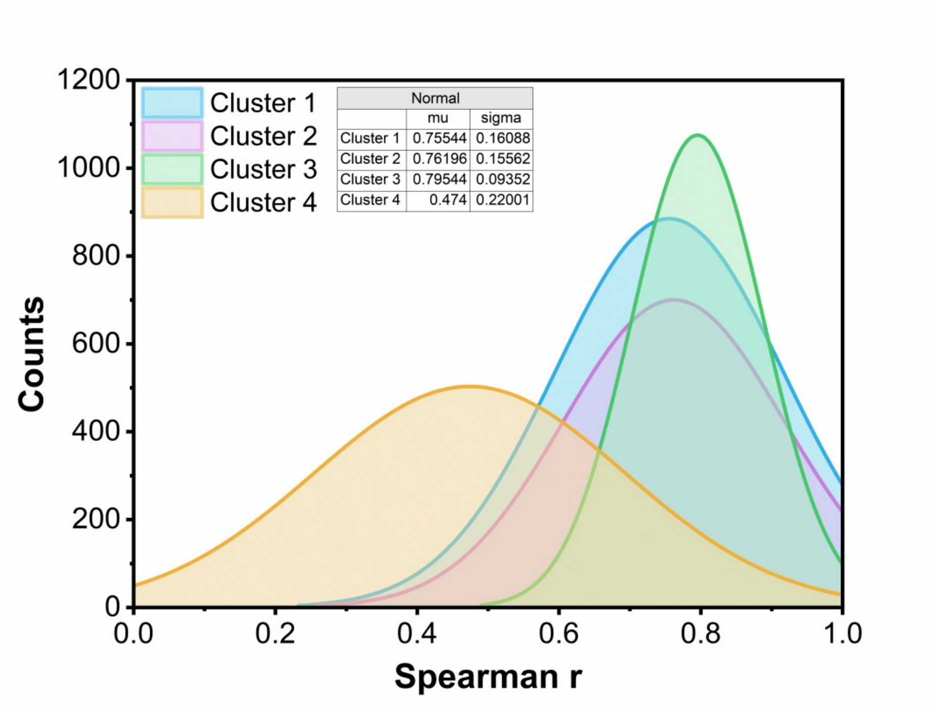

Fig. S12. Distribution of Spearman correlation coefficients obtained by comparing typical CO spectra selected from each cluster with the remaining spectra in the database.

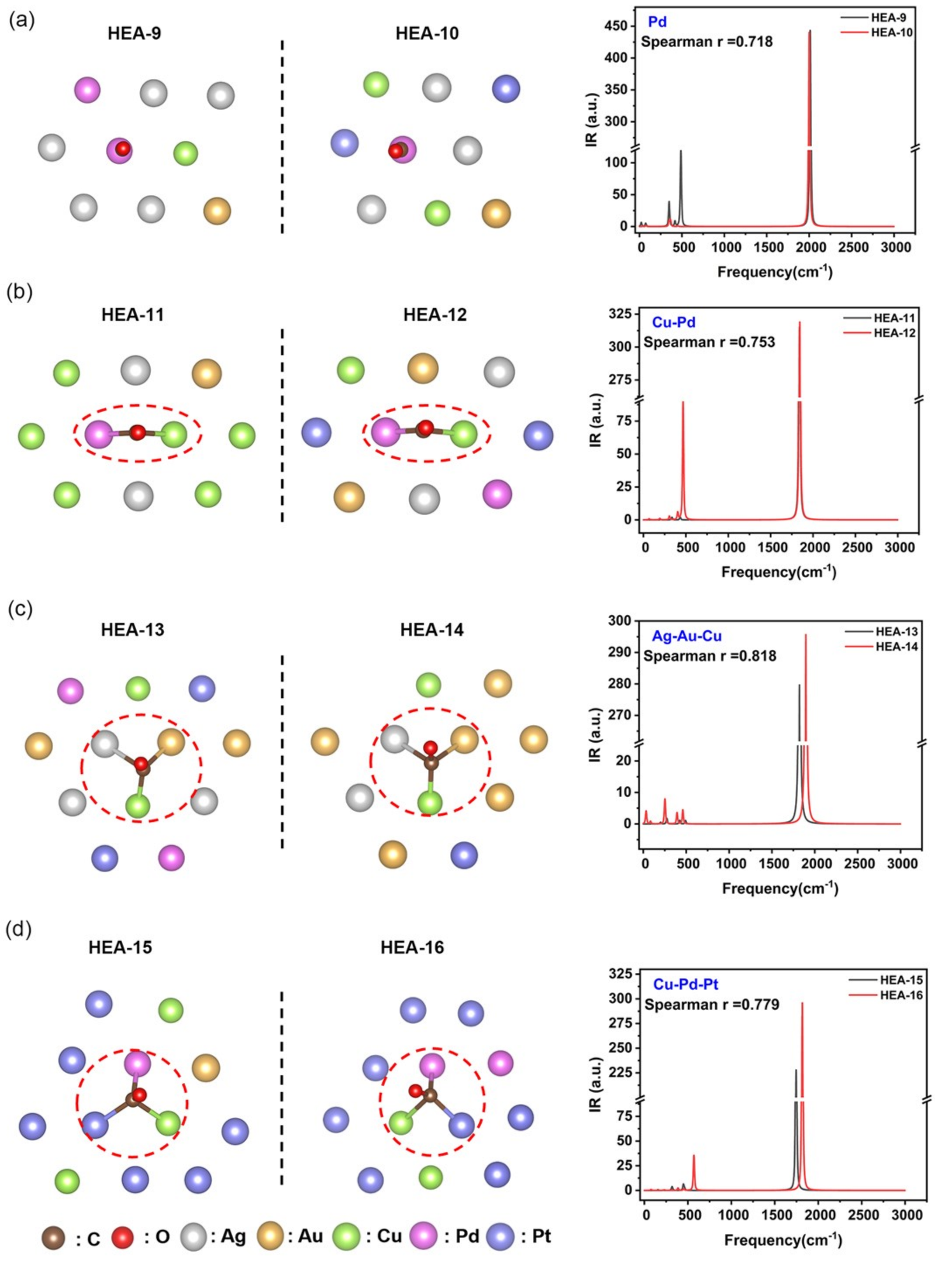

Fig. S13. (a)–(d) represent infrared spectra of CO adsorption on HEAs with identical active sites but different surrounding metal atoms, respectively.

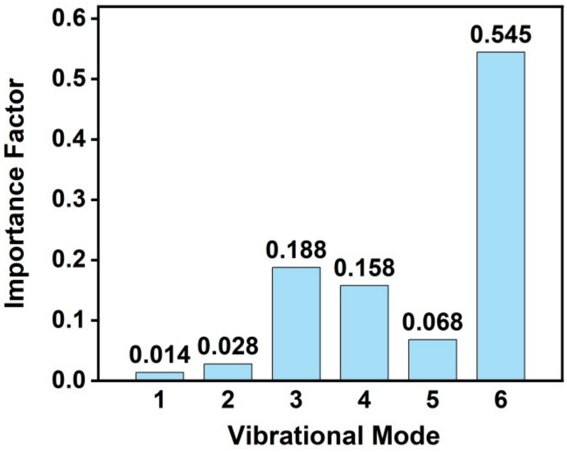

Fig. S14. Importance analysis of infrared vibrational spectra using the XGBoost method.

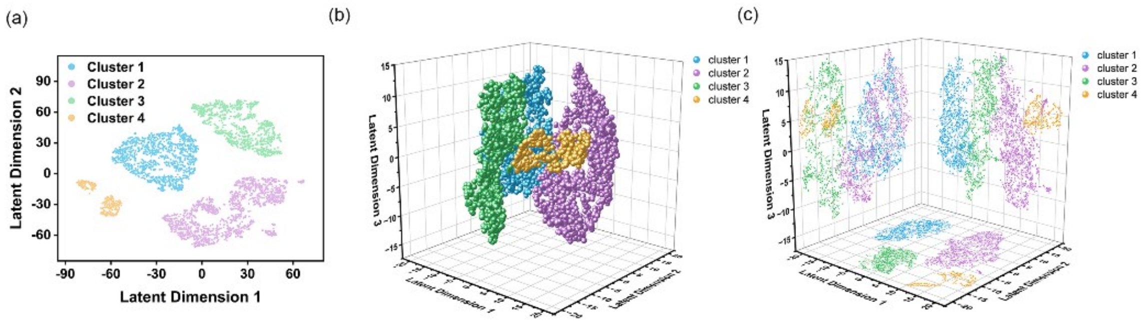

Fig. S15. (a) t-SNE 2D visualization results of the spectra. (b) t-SNE 3D visualization results of the spectra. (c) Projection of the 3D visualization onto a 2D plane.

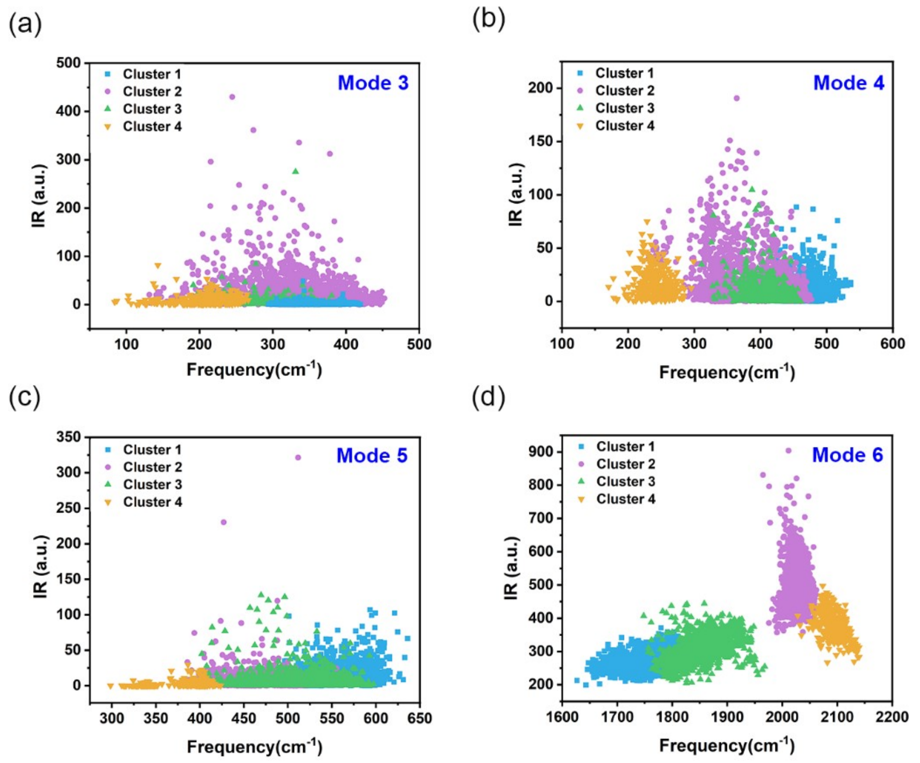

Fig. S16. (a) Distribution of frequencies and intensities for the third vibrational mode in each cluster. (b) Distribution of frequencies and intensities for the fourth vibrational mode in each cluster. (c) Distribution of frequencies and intensities for the fifth vibrational mode in each cluster. (d) Distribution of frequencies and intensities for the sixth vibrational mode in each cluster.

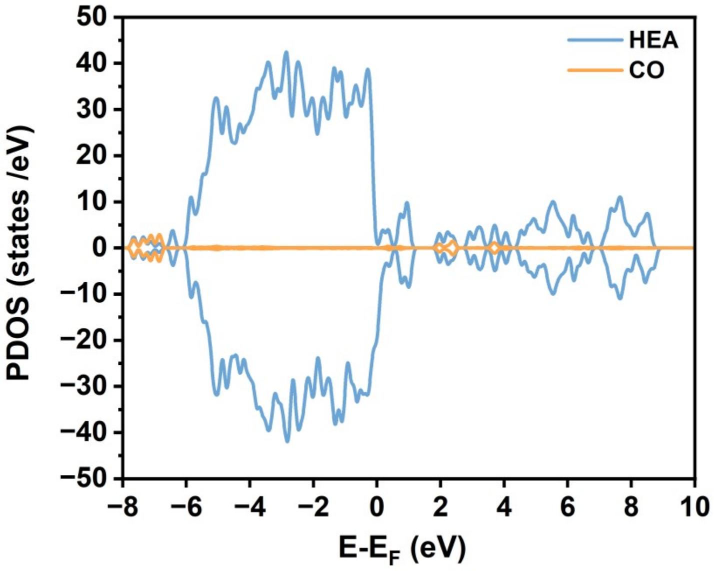

Fig. S17. The PDOS of CO and HEA when CO is adsorbed on the high-entropy alloy.

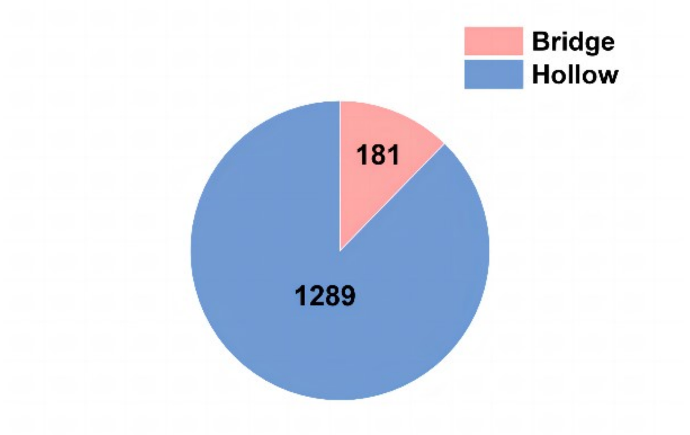

Fig. S18. Statistical analysis of CO adsorption modes in cluster 1.

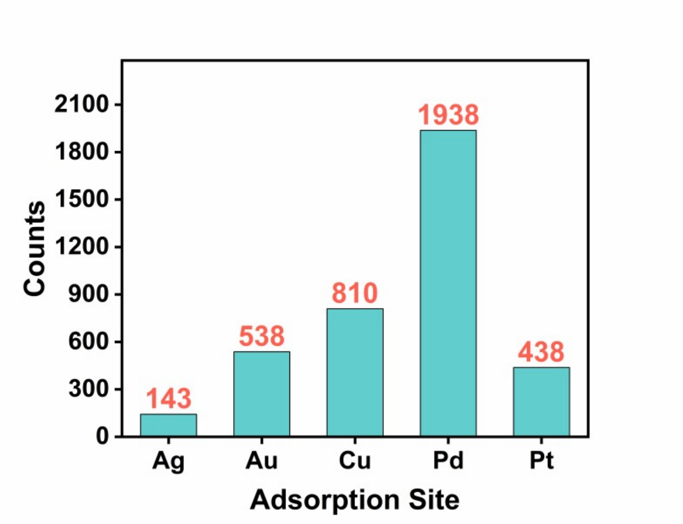

Fig. S19. Statistical analysis of CO hollow adsorption sites in cluster 1.

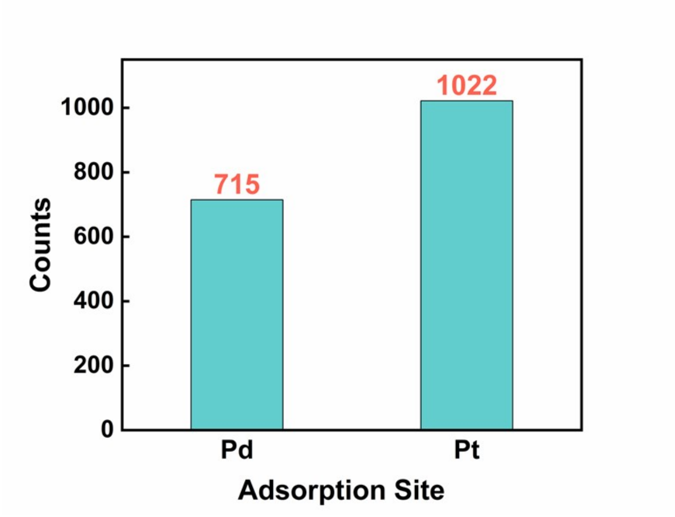

Fig. S20. Statistical analysis of CO adsorption sites in cluster 2.

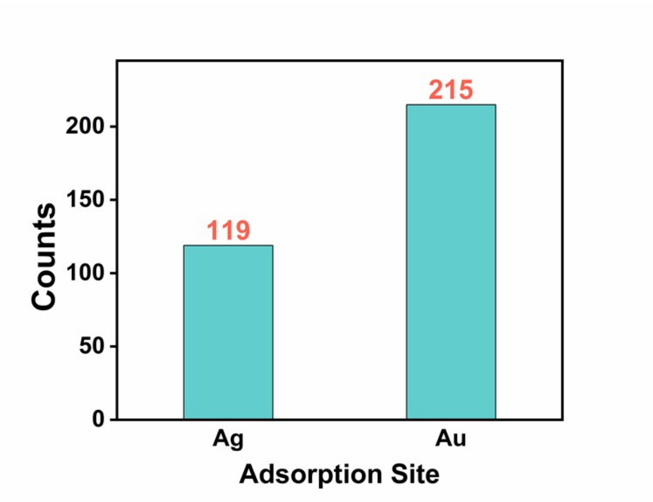

Fig. S21. Statistical analysis of CO adsorption sites in cluster 4.

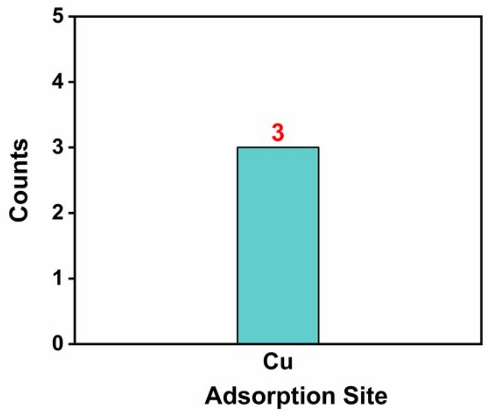

Fig. S22. Statistical analysis of CO top adsorption mode in cluster 3.

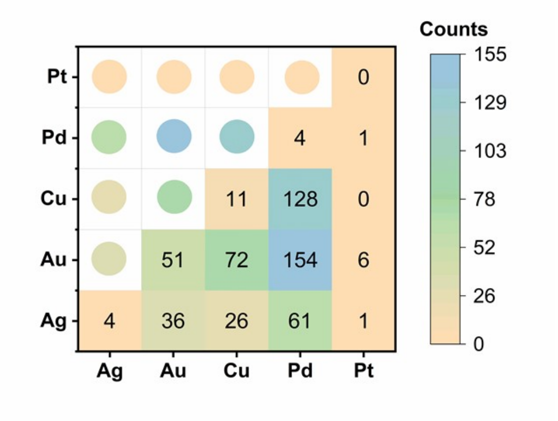

Fig. S23. Statistical analysis of CO bridge adsorption mode in cluster 3.

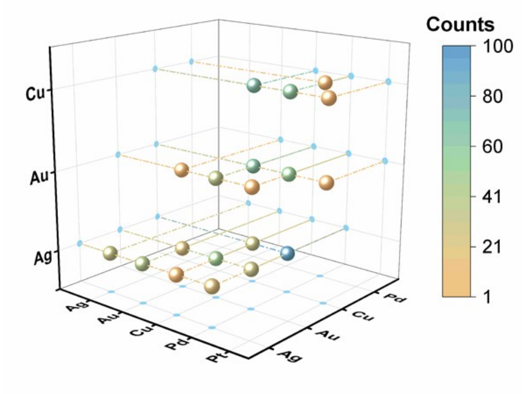

Fig. S24. Statistical analysis of CO hollow adsorption mode in cluster 3.

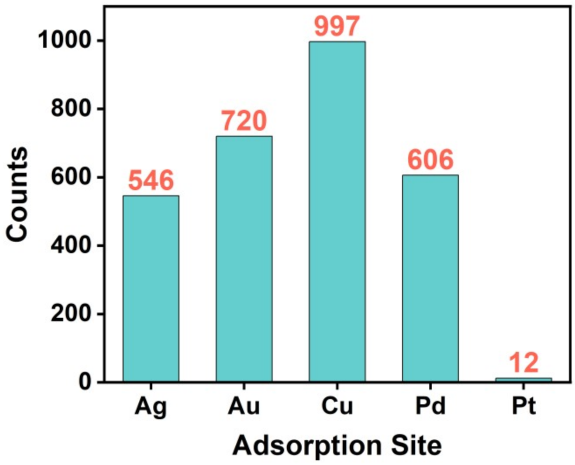

Fig. S25. Statistical analysis of CO adsorption sites in cluster 3.

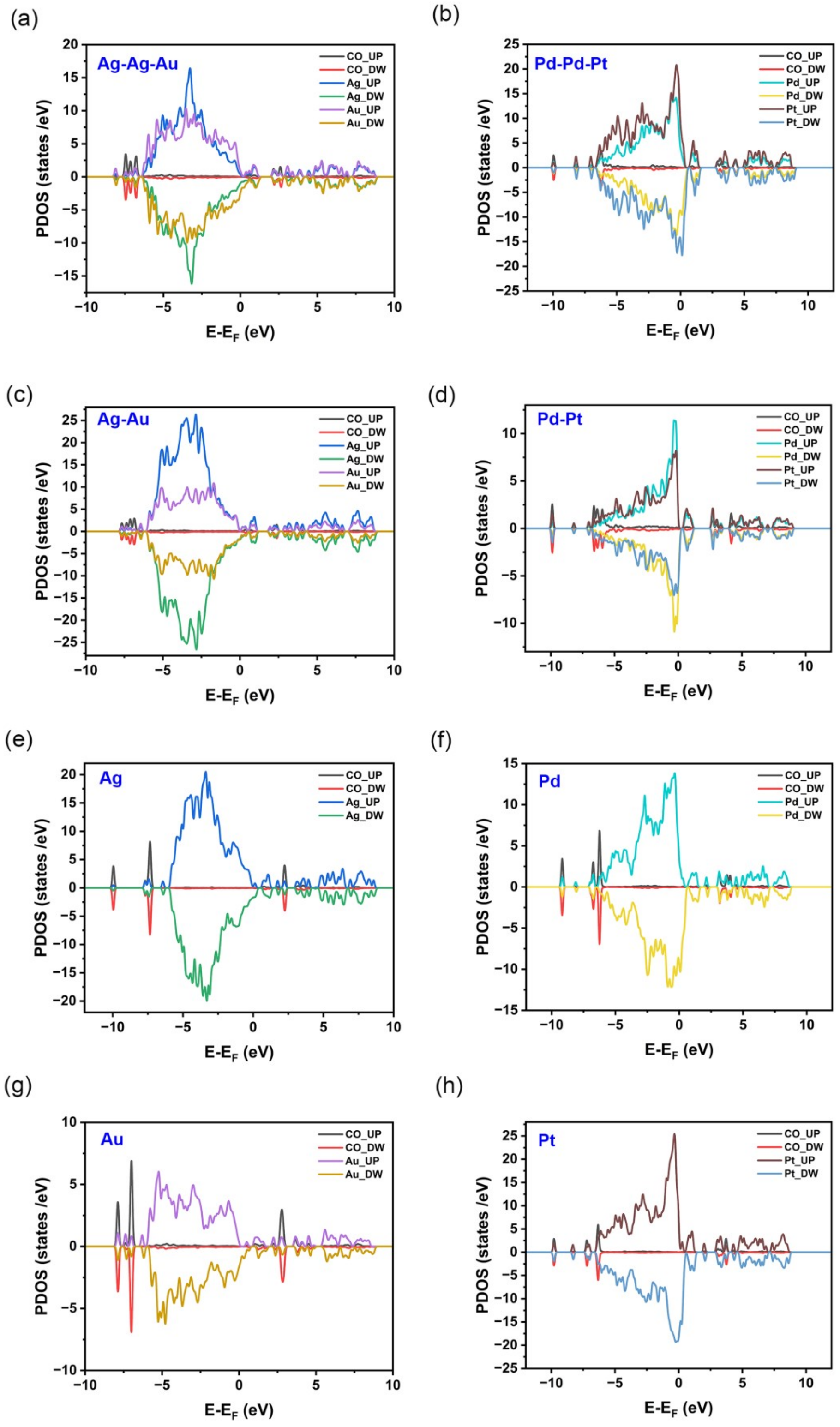

Fig. S26. (a)-(h) represent the PDOS of metal atoms and CO at different adsorption sites, respectively.

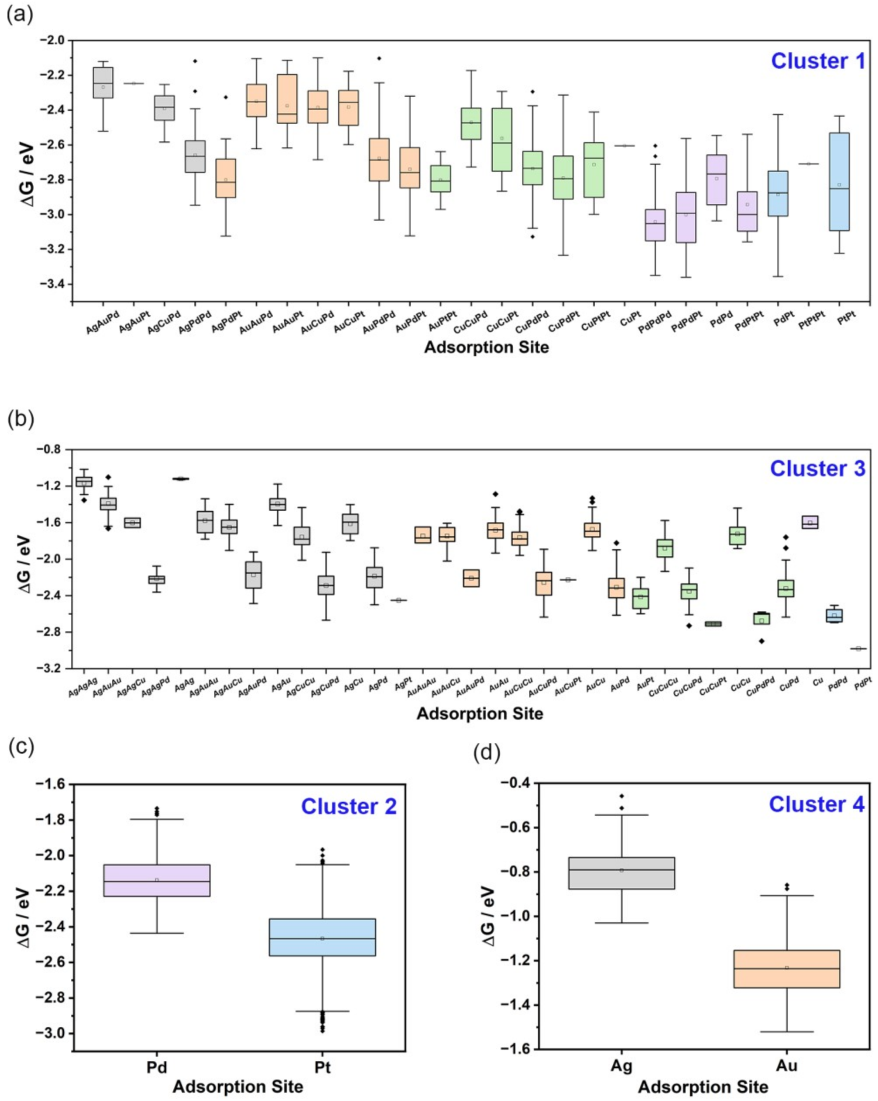

Fig. S27. (a) A box plot showing the adsorption energies for different metal compositions at specific adsorption sites within cluster 1. (b) A box plot showing the adsorption energies for different metal compositions at specific adsorption sites within cluster 3. (c) A box plot showing the adsorption energies for different metal compositions at specific adsorption sites within cluster 2. (d) A box plot showing the adsorption energies for different metal compositions at specific adsorption sites within cluster 4.

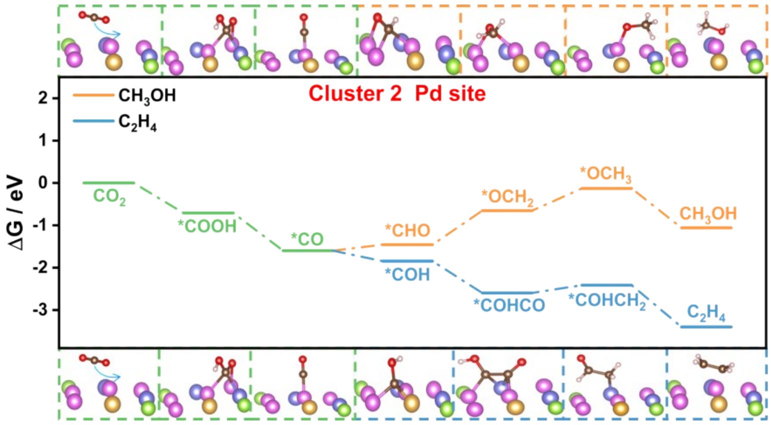

Fig. S28. Free energy diagrams for the production of CH_3OH and C_2H_4 on the Pd site in cluster 2.

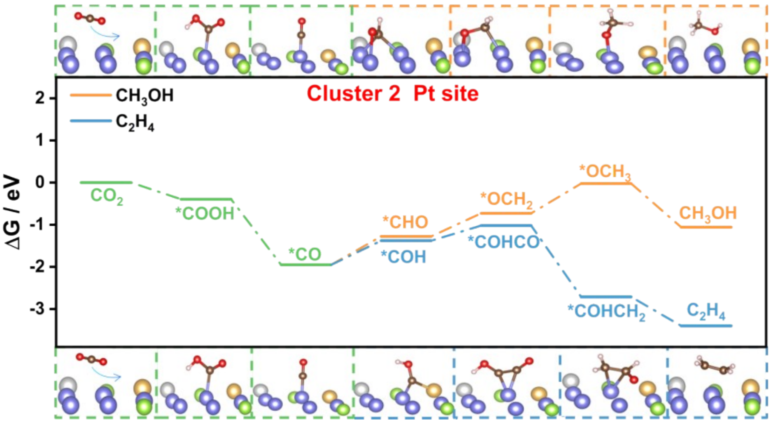

Fig. S29. Free energy diagrams for the production of CH_3OH and C_2H_4 on the Pt site in cluster 2.

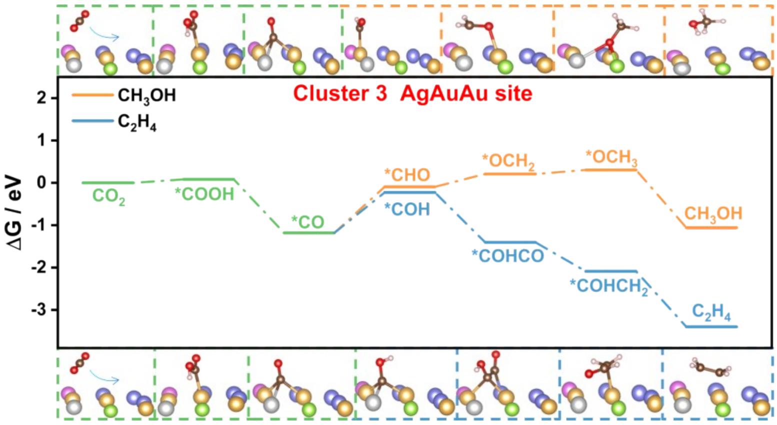

Fig. S30. Free energy diagrams for the production of CH_3OH and C_2H_4 on the hollow site composed of AgAuAu metal atoms in cluster 3.

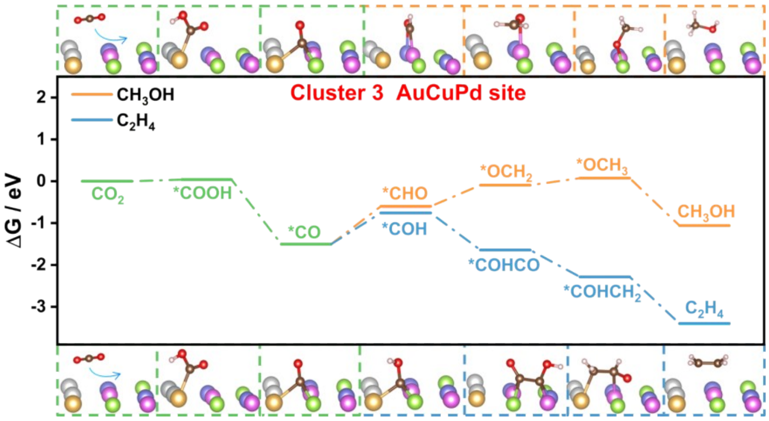

Fig. S31. Free energy diagrams for the production of CH_3OH and C_2H_4 on the hollow site composed of AuCuPd metal atoms in cluster 3.

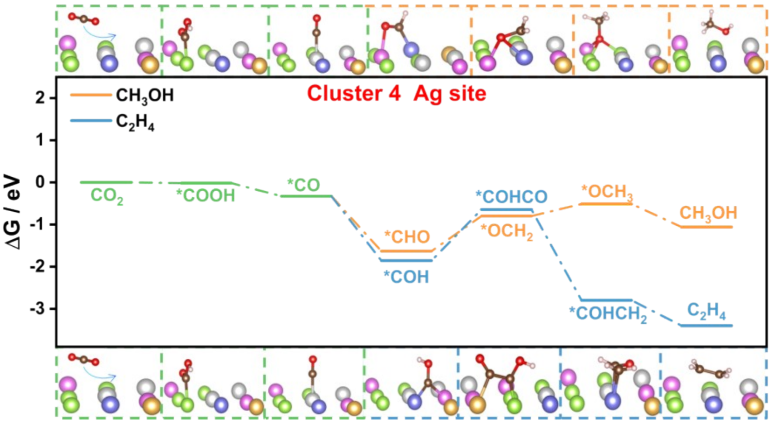

Fig. S32. Free energy diagrams for the production of CH_3OH and C_2H_4 on the Ag site in cluster 4.

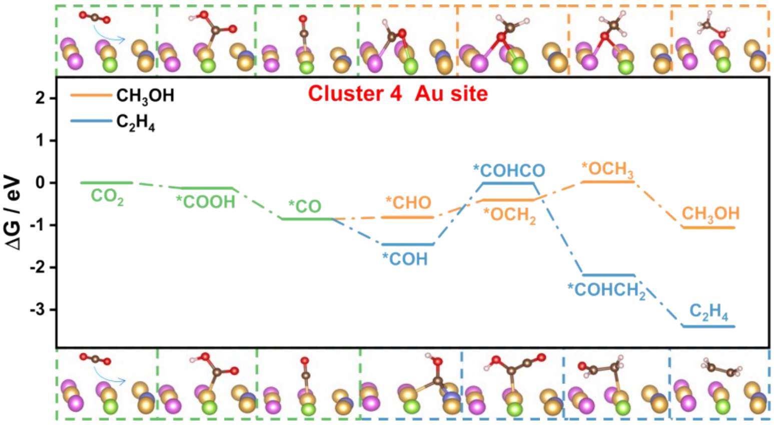

Fig. S33. Free energy diagrams for the production of CH_3OH and C_2H_4 on the Au site in cluster 4.

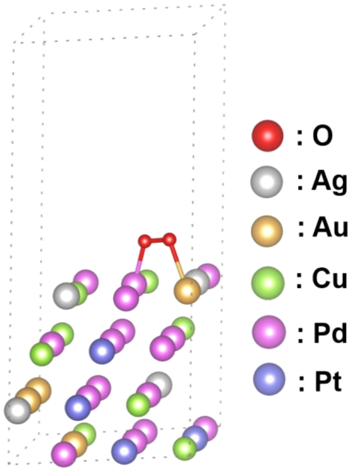

Fig. S34. The initial configuration of O_2 adsorption on the AgAuCuPdPt high-entropy alloy surface.

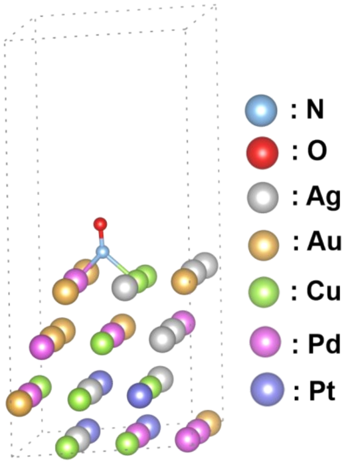

Fig. S35. The initial configuration of NO adsorption on the AgAuCuPdPt high-entropy alloy surface.

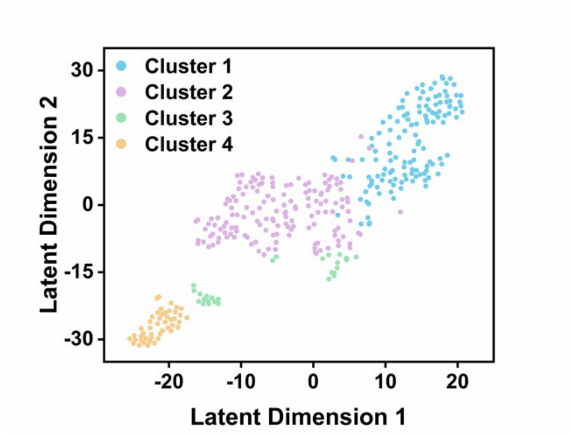

Fig. S36. Plot of the four clusters of systems with adsorbed O_2 using the t-SNE method.

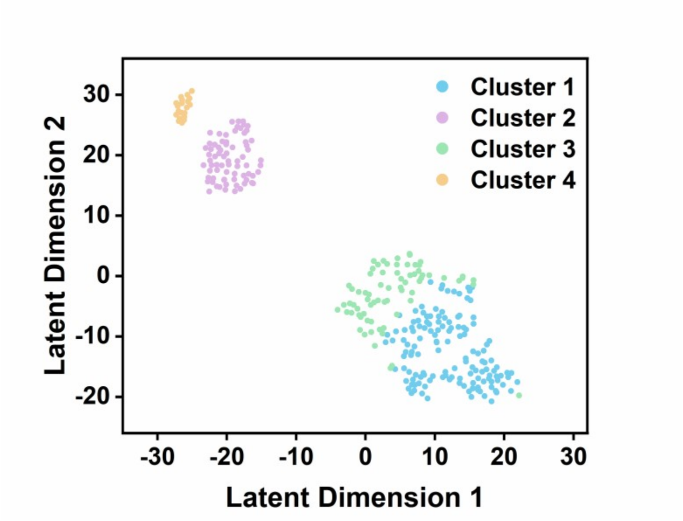

Fig. S37. Plot of the four clusters of systems with adsorbed NO using the t-SNE method.

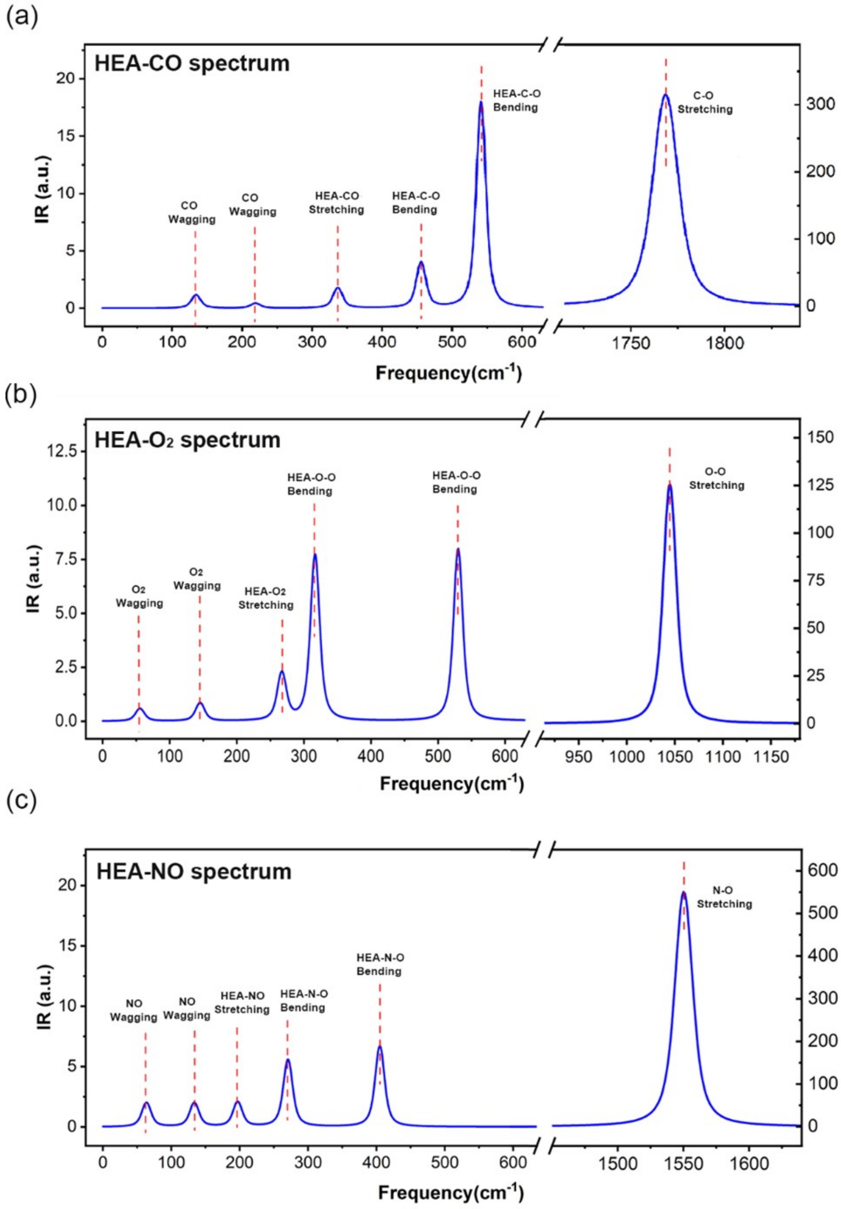

Fig. S38. (a-c) Schematic diagram of the infrared spectra of CO, O_2, and NO adsorbed on the high-entropy alloy surface.

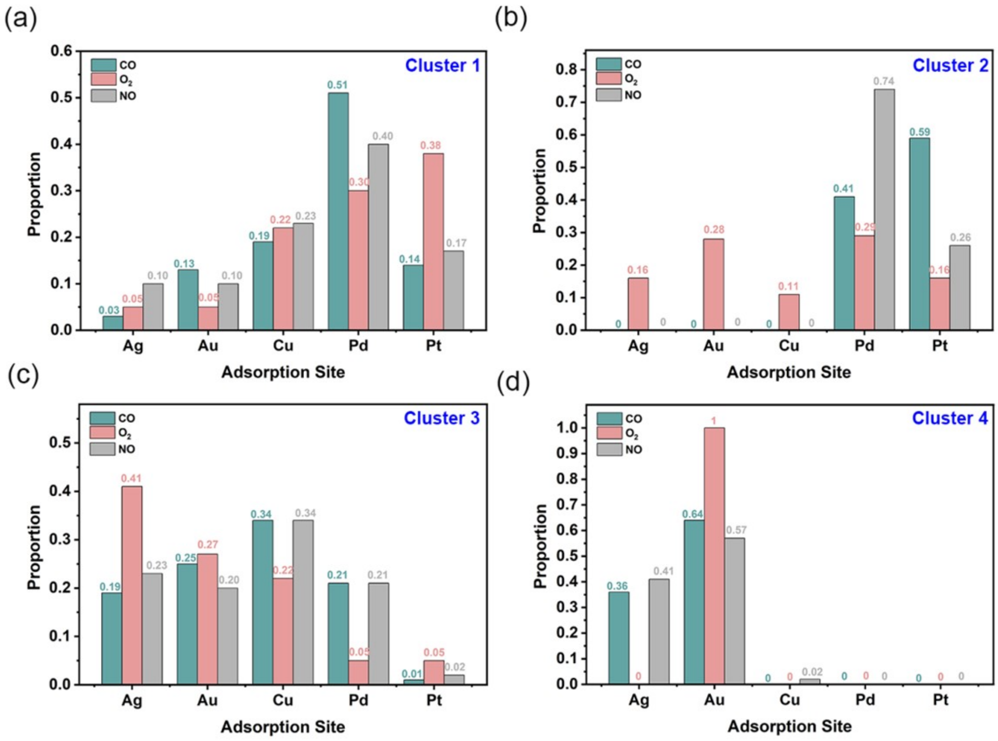

Fig. S39. (a) Proportion of each metal in the adsorption sites of CO, O_2, and NO in cluster 1. (b) Proportion of each metal in the adsorption sites of CO, O_2, and NO in cluster 2. (c) Proportion of each metal in the adsorption sites of CO, O_2, and NO in cluster 3. (d) Proportion of each metal in the adsorption sites of CO, O_2, and NO in cluster 4.

References

1. A. Van De Walle, M. Asta and G. Ceder, *Calphad*, 2002, **26**, 539-553.
2. A. Van de Walle, P. Tiwary, M. De Jong, D. Olmsted, M. Asta, A. Dick, D. Shin, Y. Wang, L.-Q. Chen and Z.-K. Liu, *Calphad*, 2013, **42**, 13-18.
3. J. K. Pedersen, T. A. Batchelor, A. Bagger and J. Rossmeisl, *ACS. Catal.*, 2020, **10**, 2169-2176.
4. G. Kresse and J. Furthmüller, *Comp. Mater. Sci.*, 1996, **6**, 15-50.
5. S. Grimme, S. Ehrlich and L. Goerigk, *J. Comput. Chem.*, 2011, **32**, 1456-1465.
6. J. P. Perdew, K. Burke and M. Ernzerhof, *Phys. Rev. Lett.*, 1996, **77**, 3865.
7. P. E. Blöchl, *Phys. Rev. B*, 1994, **50**, 17953.
8. D. Yang, X. Hu and D. Xie, *J. Comput. Chem.*, 2019, **40**, 1084-1090.
9. C. Adamo and V. Barone, *J. Chem. Phys*, 1999, **110**, 6158-6170.
10. V. A. Rassolov, J. A. Pople, M. A. Ratner and T. L. Windus, *J. Chem. Phys*, 1998, **109**, 1223-1229.
11. P. J. Hay and W. R. Wadt, *J. Chem. Phys*, 1985, **82**, 299-310.
12. W. Du, F. Ma, B. Zhang, J. Zhang, D. Wu, E. Sharman, J. Jiang and Y. Wang, *J. Am. Chem. Soc.*, 2023, **146**, 811-823.
13. L. Zhang, Y. Hou, F. Ge and P. O. Dral, *Phys. Chem. Chem. Phys.*, 2023, **25**, 23467-23476.
14. S. Kong, X. Lv, X. Wang, Z. Liu, Z. Li, B. Jia, D. Sun, C. Yang, L. Liu and A. Guan, *Nat. Catal.*, 2023, **6**, 6-15.
15. Y. Cao, S. Chen, S. Bo, W. Fan, J. Li, C. Jia, Z. Zhou, Q. Liu, L. Zheng and F. Zhang, *Angew. Chem. Int. Ed.*, 2023, **62**, e202303048.
16. T. A. Manz and N. G. Limas, *RSC Adv.*, 2016, **6**, 47771-47801.
17. C. Bouveyron and C. Brunet-Saumard, *Comput. Stat. Data Anal.*, 2014, **71**, 52-78.
18. A. P. Dempster, N. M. Laird and D. B. Rubin, J. R. Stat. Soc. B., 1977, **39**, 1-22.
